# *Monopis jussii*, a new species (Lepidoptera, Tineidae) inhabiting nests of the Boreal owl (*Aegolius funereus*)

**DOI:** 10.3897/zookeys.992.53975

**Published:** 2020-11-12

**Authors:** Marko Mutanen, Peter Huemer, Jonna Autto, Ole Karsholt, Lauri Kaila

**Affiliations:** 1 Ecology and Genetics Research Unit, P.O.Box 3000, FI-90014 University of Oulu, Finland University of Oulu Oulu Finland; 2 Tiroler Landesmuseen-Betriebsgesellschaft m.b.H., Innsbruck, Austria Tiroler Landesmuseen-Betriebsgesellschaft m.b.H. Innsbruck Austria; 3 Apajatie 11, FI-96800 Rovaniemi, Finland Unaffiliated Rovaniemi Finland; 4 Zoological Museum, Natural History Museum of Denmark, Universitetsparken 15, DK-2100 Copenhagen, Denmark Natural History Museum of Denmark Copenhagen Denmark; 5 Finnish Museum of Natural History, Zoology Unit, P.O.Box 17, FI-00014 University of Helsinki, Finland University of Helsinki Helsinki Finland

**Keywords:** boreo-montane, cryptic diversity, DNA barcoding, nuclear marker

## Abstract

*Monopis jussii* Kaila, Mutanen, Huemer, Karsholt & Autto, **sp. nov.** (Lepidoptera, Tineidae) is described as a new species. It is closely related to the widespread and common *M. laevigella* ([Denis & Schiffermüller], 1775), but differs in its distinct COI DNA barcode sequences, four examined nuclear loci as well as details in forewing coloration and pattern. Most reared specimens of *M. jussii* have emerged from the nest remnants of the Boreal owl (*Aegolius funereus* (Linnaeus, 1758)), but also nests of the Ural owl (*Strix uralensis* Pallas, 1771) and the Great tit (*Parus major* Linnaeus, 1758) have been observed as suitable habitats. Based on the present knowledge, the new species has a boreo-montane distribution as it is recorded only from northern Europe and the Alps. Several extensive rearing experiments from *Strix* spp. nest remnants from southern Finland did not produce any *M. jussii*, but thousands of *M. laevigella*, suggesting that the species is lacking in the area or, more unlikely, that the nest of these owl species do not serve as good habitat for the new species. This unexpected species discovery highlights, once again, the usefulness of DNA barcoding in revealing the cryptic layers of biodiversity. To serve stability we select a neotype for *Tinea laevigella* [Denis & Schiffermüller], 1775, and discuss the complicated synonymy and nomenclature of this species.

## Introduction

The lepidopteran fauna of Central and North Europe has been investigated for a longer time and more intensively than that of any other region in the world. Consequently, discoveries of species new to the region are nowadays uncommon and usually involve expansive or invasive species. Large-scale efforts to build taxonomically comprehensive regional DNA barcode reference libraries have, however, resulted in a boost in discoveries of overlooked species during the last 15 years, as demonstrated by the increase of new species descriptions e.g. in the family Gelechiidae by Huemer et al. (2020). Characteristic to the new discoveries is that they often concern unexpected cases of cryptic diversity among well-known and often widespread species. Examples of such recent findings, originally detected as deep intraspecific splits in DNA barcode sequences, include *Leptidea reali* Reissinger, 1990 (Dinca et al. 2011), *Olethreutes subtilana* (Falkovitsh, 1959) (Segerer et al. 2010), *Phalonidia udana* (Guenée, 1845) (Mutanen et al. 2012a), *Epinotia cinereana* (Haworth, 1811) (Mutanen et al. 2012b), *Nemophora scopolii* Kozlov, Mutanen, Lee & Huemer, 2016 (Kozlov et al. 2017), several *Elachista* spp. (Mutanen et al. 2013) and *Hoplodrina alsinides* (Costantini, 1922) (Huemer et al. 2020).

There are many more additional cases of potential cryptic diversity in European Lepidoptera, as dozens of species show high levels of genetic polymorphism in their mitochondrial DNA (Mutanen et al. 2016, Huemer et al. 2020). While polymorphism in the mitochondrial DNA may result from multiple other phenomena, including mitochondrial introgression and retained ancestral polymorphism, many of those cases are likely to result from cryptic diversity.

An intraspecific split of the mitochondrial DNA being reflected in the nuclear genome in sexually reproducing species and in sympatry would strongly suggest the presence of cryptic diversity, because, unlike mitochondrial DNA, nuclear DNA is subject to genetic recombination. From this starting point, we sequenced four nuclear markers of *Monopis laevigella* ([Denis & Schiffermüller], 1775), a widespread and common species of tineid moths, showing a deep sympatric genetic split in its DNA barcode region in Europe (Gaedike 2019). Despite the limited number of analyzed specimens, the results provided unequivocal genetic support for the presence of two biologically distinct species. Subsequent morphological examination revealed consistent differences in the adult wing patterns, providing additional support for the overlooked cryptic diversity. Additionally, based on the presently available data, the two species show overlapping, but different ranges and based on the present knowledge, also a different ecology. Based on these grounds, we here describe one of the taxa as new to science.

## Material and methods

The material examined was acquired from the following collections:

**ITJ** Research collection of Juhani Itämies

**MUT** Research collection of Marko & Tomi Mutanen

**MZH** Finnish Museum of Natural History, Helsinki, Finland

**TLMF** Tiroler Landesmuseum Ferdinandeum, Innsbruck, Austria

**ZMUO** Zoological Museum, University of Oulu, Finland

**ZSM** Zoologische Staatssammlung München, Germany

Terminology of genitalia follows Robinson and Nielsen (1993) and Gaedike (2019).

Preparation of genitalia generally follows the method outlined by Robinson (1976). Male genitalia were mounted in dorso-ventral position as it was considered to best show shapes of diagnostic structures, even if the shape of the gnathos is not optimally expressed. Male genitalia were stained using Eosin, female genitalia as well as abdominal pelts of both sexes using Chlorazol black. Structures were embedded in Euparal. Images were edited using Corel PHOTO-PAINT (2019).

Species of Tineidae have been systematically sequenced for the standard barcode region of the mitochondrial COI (cytochrome c oxidase subunit 1) in the connection of ongoing regional or national DNA barcoding projects in the Alps (Lepidoptera of the Alps campaign) and Finland (FinBOL). DNA barcode sequencing was conducted at the Canadian Centre for DNA Barcoding (CCDB, Biodiversity Institute of Ontario, University of Guelph) using standard Sanger protocols as explained in deWaard et al. (2008). We successfully sequenced 87 specimens of *Monopis* representing twelve species, the newly described species included. Five European species of *Monopis* (*M. luteocostalis* Gaedike, 2006, *M. henderickxi* Gaedike & Karsholt, 2001, *M. christophi* Petersen, 1957, *M. pallidella* Zagulajev, 1955 and *M. barbarosi* (Koçak, 1981)) were not included in this sampling. Each of them is morphologically clearly distinct from *M. jussii* sp. nov. (Gaedike 2019). Full collection and taxonomic data as well as voucher photographs, DNA sequences and GenBank accession numbers of all these specimens are available in the Barcode of Life Data Systems (BOLD; Ratnasingham and Hebert 2007) in the public dataset DS-MONOJUS at https://dx.doi.org/10.5883/DS-MONOJUS. Collection data of the specimens are also given in Table 1. Some of the COI sequences used in this study were previously published in Mutanen et al. (2016), the others are novel.

**Table 1.** Summary of the collection data of barcoded specimens of *Monopis* used in this study. For more details, see the public BOLD dataset at https://dx.doi.org/10.5883/DS-MONOJUS.

Species	Sample ID	Sequence length	Collector(s)	Collection date	Country	Province	Site	Latitude / Longitude
*Monopis burmanni*	TLMF Lep 18816	658	Huemer P.	13-Jun-2006	Austria	Tyrol	Nordtirol, Kranebitter Innaue	47.265, 11.323
*Monopis burmanni*	TLMF Lep 18234	658	Huemer P.	05-Jun-2015	Austria	Tyrol	Nordtirol, Ellbachtal, unterer Kaiserboden	47.539, 11.926
*Monopis crocicapitella*	TLMF Lep 06512	658	O. Rist	23-Sep-2005	Austria	Vienna	Wien Stadlau	48.217, 16.467
*Monopis crocicapitella*	TLMF Lep 03882	658	Huemer P.	21-May-2004	Spain	Comunidad Valenciana	Valencia, El Saler, Albufera	39.3255 -0.312972
*Monopis fenestratella*	MM18616	658	Marko Mutanen	1997	Finland	N	Mäntsälä	60.688, 25.168
*Monopis fenestratella*	MM18615	658	Marko Mutanen	1997	Finland	N	Mäntsälä	60.688, 25.168
*Monopis fenestratella*	MM08511	658	Marko Mutanen	larva 1997-1998	Finland	Ta	Pälkäne		
*Monopis fenestratella*	MM08510	552	Marko Mutanen	larva 1997-1998	Finland	Ta	Pälkäne		
*Monopis imella*	TLMF Lep 19836	658	Buchner P.	29-Aug-2014	Austria		Niederoesterreich, Sollenau	47.905, 16.266
*Monopis imella*	TLMF Lep 25734	639	Huemer P.	07-Sep-2016	Austria		Burgenland, Jois SW, Hackelsberg	47.9539, 16.7747
*Monopis imella*	TLMF Lep 25735	638	Huemer P.	07-Sep-2016	Austria		Burgenland, Jois SW, Hackelsberg	47.9539, 16.7747
*Monopis imella*	TLMF Lep 23122	658	Huemer P.	26-May-2017	Austria		Burgenland, Hackelsberg	47.9528, 16.7733
*Monopis imella*	TLMF Lep 19838	658	Buchner P.	17-Aug-2014	Austria		Niederoesterreich, Sollenau	47.905, 16.266
*Monopis imella*	MM18899	658	Kari Vaalamo, Bo Wikström	13-Jul-2002-19-Jul-2002	Finland	Al	Kökar	59.9031, 20.74
*Monopis imella*	MM18898	658	Pekka Sundell, M. Varesvuo, L. Jalonen, Kalle Lundsten	25-Aug-2004-10-Sep-2004	Finland	Al	Kökar	59.92, 20.898
*Monopis imella*	MM26020	658	Huotari, Laasonen	08-Jul-2014	Hungary	Tokaj	Tarcal	48.0512, 21.1811
*Monopis imella*	MM26021	658	Huotari, Laasonen	08-Jul-2014	Hungary		Tokaj, Tarcal	48.0512, 21.1811
*Monopis jussii*	MM17525	658	Marko Mutanen	2001	Finland	Oba	Ylikiiminki	64.984, 26.153
*Monopis jussii*	MM18626	658	Panu Välimäki & Marko Mutanen	2006	Finland	Oba	Oulu	64.9768, 25.3056
*Monopis jussii*	MM15526	658	Marko Mutanen	larva 2001	Finland	Oba	Ylikiiminki		
*Monopis jussii*	TLMF Lep 09795	658	Huemer P.	23-Jun-2006	Italy	South Tyrol	Suedtirol, Tiers E, Plafetscher Wald	46.472, 11.596
*Monopis laevigella*	TLMF Lep 09306	658	Huemer P.	19-Jun-2012	Austria	Tyrol	Nordtirol, Oberpettnau, Platten	47.301, 11.126
*Monopis laevigella*	TLMF Lep 10365	658	Huemer P.	16-Jun-2013	Austria	Tyrol	Nordtirol, Tiefenbachklamm/ Brandenberg	47.484, 11.864
*Monopis laevigella*	TLMF Lep 10441	658	Huemer P.	16-May-2013	Austria	Tyrol	Nordtirol, Tiefenbachklamm/ Brandenberg	47.484, 11.864
*Monopis laevigella*	TLMF Lep 07389	658	Huemer P.	25-May-2008	Austria	Tyrol	Nordtirol, Telfs/ Moritzen SW, Innau	47.299, 11.05
*Monopis laevigella*	TLMF Lep 10354	658	Huemer P.	16-Jun-2013	Austria	Tyrol	Nordtirol, Tiefenbachklamm/ Brandenberg	47.484, 11.864
*Monopis laevigella*	TLMF Lep 07970	658	Huemer P.	25-May-2012	Austria	Vorarlberg	Umg.Zwischenwasser, Ueble Schlucht, Eingang	47.267, 9.667
*Monopis laevigella*	MM19355	658	O. Martin	larva 14-Oct-2004	Denmark	Sjaelland	Nez, Bognaes, Egehoved		
*Monopis laevigella*	MM17303	658	Tomi Mutanen	09-Jun-2010	Finland	Ab	Salo	60.335, 23.088
*Monopis laevigella*	MM17522	658	Henrik Bruun	01-Apr-2007	Finland	Ab	Nauvo	60.225, 21.945
*Monopis laevigella*	MM21029	658	Ali Karhu	27-Jun-2008-29-Jun-2008	Finland	Ka	Liperi	62.552, 29.167
*Monopis laevigella*	MM21028	658	Ali Karhu	1-Jun-2010-25-Jul-2010	Finland	Ka	Liperi	62.551, 29.226
*Monopis laevigella*	MM21026	658	Ali Karhu	03-Jul-2007	Finland	Ka	Liperi	62.563, 29.013
*Monopis laevigella*	MM21025	658	Ali Karhu	2005	Finland	Ka	Liperi	62.511, 29.475
*Monopis laevigella*	MM17524	606	Marko Mutanen	30-Jun-1997	Finland	Oba	Hailuoto	64.968, 24.671
*Monopis laevigella*	MM15527	658	Marko Mutanen	30-Jun-2001	Finland	Oba	Oulu	64.977, 25.306
*Monopis laevigella*	MM10119	658	Marko Mutanen, Nestori Mutanen, Anttoni Mutanen	12-Jul-2008	Finland	Oba	Kiiminki	65.071, 25.725
*Monopis laevigella*	MM18625	658	Panu Välimäki	21-Jun-2000	Finland	St	Luvia	61.29, 21.587
*Monopis laevigella*	MM17526	658	Juhani Itaemies	14-Feb-2005	Finland	St	Eurajoki	61.193, 21.417
*Monopis laevigella*	TLMF Lep 27537	658	Huemer P.	29-Jun-2019	Italy	Piedmont	Fenestrelle, ca. 0,7 km NE Pequerel	45.0517, 7.07111
*Monopis laevigella*	TLMF Lep 12113	658	Huemer P.	17-Jul-2013	Italy	South Tyrol	Suedtirol, N Zwischenwasser/ St. Lorenzen	46.739, 11.873
*Monopis laevigella*	TLMF Lep 11818	658	Huemer P.	25-Jul-2013	Italy	South Tyrol	Suedtirol, Franzenshoehe / Stilfserjoch	46.534, 10.486
*Monopis laevigella*	TLMF Lep 02066	658	Huemer P.	01-Jul-2010	Italy	South Tyrol	Suedtirol, Ritten/ Obergruenwald	46.597, 11.439
*Monopis laevigella*	TLMF Lep 05368	658	Huemer P., Tarmann G. M.	01-Aug-2011	Macedonia		Mavrovo NP, Radika valley, around bridge, 10 km NNW Sveta Voda	41.789, 20.547
*Monopis monachella*	TLMF Lep 08436	658	Huemer P.	25-Jul-2012	Austria	Vorarlberg	Lustenau, Schweizer Ried, AZE Haeusle S	47.446, 9.69
*Monopis monachella*	TLMF Lep 19839	658	Buchner P.	07-Jun-2014	Austria		Niederoesterreich, Sollenau	47.905, 16.266
*Monopis monachella*	MM13366	658	Marko Mutanen, Panu Välimäki	2008	Finland	Ab	Dragsfjärd	60.011, 22.498
*Monopis monachella*	MM11934	658	Marko Mutanen, Panu Välimäki	2007	Finland	N	Hanko	59.836, 23.236
*Monopis monachella*	MM17249	658	Lauri Kaila	21-Aug-2005	Finland	N	Tammisaari	59.829, 23.612
*Monopis monachella*	MM12377	658	Marko Mutanen, Panu Välimäki	2007	Finland	Sa	Imatra	61.108, 28.799
*Monopis neglecta*	TLMF Lep 07250	658	Sumpich J.	10-Jun-2010	Austria	Lower Austria	Hardegg Umgebung/ Thaya Haenge	48.854, 15.858
*Monopis neglecta*	TLMF Lep 17583	658	Deutsch H.	30-Aug-2002	Austria	Tyrol	Osttirol, Lengberg	46.801, 12.891
*Monopis neglecta*	TLMF Lep 06608	658	Rist O.	11-Jun-2010	Austria	Vienna	Wien Mauer	48.15, 16.25
*Monopis nigricantella*	TLMF Lep 03881	658	Huemer P.	07-Sep-2005	Spain	Comunidad Valenciana	Valencia, El Saler, Albufera	39.3255, -0.312972
*Monopis nigricantella*	TLMF Lep 03879	658	Huemer P.	18-May-2004	Spain	Comunidad Valenciana	Valencia, El Saler, Albufera	39.3255, -0.312972
*Monopis nigricantella*	TLMF Lep 03878	658	Huemer P.	22-May-2004	Spain	Comunidad Valenciana	Valencia, Santa Pola, Playa del Pinet	38.1583, -0.625278
*Monopis nigricantella*	TLMF Lep 03880	658	Huemer P.	08-Sep-2005	Spain	Comunidad Valenciana	Valencia, El Saler, Albufera	39.3255, -0.312972
*Monopis obviella*	TLMF Lep 15096	636	Huemer P.	19-Jun-2014	Austria	Tyrol	Nordtirol, Baumkirchen W	47.296, 11.552
*Monopis obviella*	TLMF Lep 09367	658	Huemer P.	02-Jun-2012	Austria	Tyrol	Nordtirol, Flaurling NW, Innau	47.302, 11.121
*Monopis obviella*	TLMF Lep 08054	658	Huemer P.	15-Jun-2012	Austria	Vorarlberg	Bludesch, Bludescher Magerrasen E, Umg. Jordan	47.203, 9.747
*Monopis obviella*	TLMF Lep 09962	658		19-Jun-13	Austria	Vorarlberg	Umg.Langenegg, Langenegg-Leiten, Fohren	47.467, 9.883
*Monopis obviella*	TLMF Lep 25739	658	Huemer P.	07-Sep-2016	Austria		Burgenland, Jois SW, Hackelsberg	47.9539, 16.7747
*Monopis obviella*	TLMF Lep 19832	658	Buchner P.	29-Aug-2014	Austria		Niederoesterreich, Sollenau	47.905, 16.266
*Monopis obviella*	MM18928	658	Kari Vaalamo, Bo Wikström	19-Jul-2008-23-Jul-2008	Finland	Al	Lemland	59.9564, 20.0116
*Monopis obviella*	MM06790	658	Marko Mutanen	13-Jul-2007	Finland	Al	Lemland	60.026, 19.961
*Monopis obviella*	MM21130	658	Marko Mutanen, Tomi Mutanen, Anttoni Mutanen, Nestori Mutanen	16-Jul-2011	Finland	N	Hanko	59.834, 23.013
*Monopis obviella*	TLMF Lep 27604	658	Huemer P.	28-Jun-2019	Italy	Piedmont	Fenestrelle, ca. 1 km WNW Pequerel	45.0497, 7.05139
*Monopis obviella*	TLMF Lep 27794	630	Huemer P.	23-Jul-2019	Italy	Piedmont	Fenestrelle, ca. 0,7 km NE Pequerel	45.0517, 7.07111
*Monopis obviella*	TLMF Lep 10292	658	Huemer P.	25-Jun-2013	Italy	South Tyrol	Suedtirol, Margreid/ Fennerschlucht	46.288, 11.201
*Monopis obviella*	TLMF Lep 02169	658	Huemer P.	04-Jun-2010	Italy	South Tyrol	Suedtirol, Montiggl/ Kleiner Priol	46.428 11.03
*Monopis obviella*	TLMF Lep 12282	658	Huemer P.	05-Jul-2013	Italy	South Tyrol	Suedtirol, Schleiser Leiten	46.698, 10.517
*Monopis spilotella*	MM04157	658	Marko Mutanen		Finland	Le	Enontekiö	68.997, 20.744
*Monopis spilotella*	MM24137	658	Marko Mutanen, Anttoni Mutanen, Nestori Mutanen	05-Jul-2014	Finland	Lkoc	Muonio	67.9178, 23.7466
*Monopis spilotella*	MM03158	658	Marko Mutanen	2006	Finland	Oba	Kiiminki	65.071, 25.725
*Monopis spilotella*	MM02304	658	Marko Mutanen, Panu Välimäki	2006	Finland	Sa	Imatra	61.108, 28.799
*Monopis weaverella*	TLMF Lep 15166	658	Huemer P.	09-Jun-2014	Austria	Tyrol	Nordtirol, Ellbachtal, unterer Kaiserboden	47.539, 11.926
*Monopis weaverella*	TLMF Lep 15178	658	Huemer P.	09-Jun-2014	Austria	Tyrol	Nordtirol, Ellbachtal, unterer Kaiserboden	47.539, 11.926
*Monopis weaverella*	TLMF Lep 18561	658	Huemer P.	20-Jul-2005	Austria	Tyrol	Nordtirol, Umg. Innsbruck, Samertal, Jaegerkar	47.34, 11.382
*Monopis weaverella*	TLMF Lep 07388	658	Huemer P.	25-May-2008	Austria	Tyrol	Nordtirol, Telfs/ Moritzen SW, Innau	47.299, 11.05
*Monopis weaverella*	TLMF Lep 09220	658	Huemer P.	06-Jun-2010	Austria	Tyrol	Nordtirol, Walchsee/ Schwemm N	47.661, 12.298
*Monopis weaverella*	MM21138	658	Marko Mutanen ,Tomi Mutanen	18-Jun-2011	Finland	Ab	Nauvo	60.192, 21.923
*Monopis weaverella*	MM13581	658	Marko Mutanen, Panu Välimäki	2008	Finland	Ab	Dragsfjärd	60.011, 22.498
*Monopis weaverella*	MM21027	658	Ali Karhu	21-Jun-2004-23-Jun-2004	Finland	Ka	Liperi	62.552, 29.167
*Monopis weaverella*	MM04159	658	Marko Mutanen		Finland	Le	Enontekiö	68.997, 20.744
*Monopis weaverella*	MM04158	658	Marko Mutanen		Finland	Le	Enontekiö	68.997, 20.744
*Monopis weaverella*	MM02600	639	Marko Mutanen, Panu Välimäki	2006	Finland	Sa	Imatra	61.108, 28.799
*Monopis weaverella*	TLMF Lep 22008	658	Schaefer W.	07-Aug-2015	Germany		Kefenrod	50.35, 9.21667

Four nuclear genes, *carbamoylphosphate synthase domain protein* (CAD), *elongation factor 1 alpha* (EF-1a), *cytosolic malate dehydrogenase* (MDH) and *wingless*, were sequenced at the University of Oulu, Finland. These genes were chosen primarily based on the high amplification success rate in other Tineidae, but also based on our previous experience on their general good functionality to provide useful taxonomic information between closely related species. In these analyses, three specimens of *M. laevigella* and two specimens of *M. jussii*, all collected from Finland, were included. Legs of dry and pinned adult specimens were used for extraction of genomic DNA with DNeasy Blood & Tissue Kit (Qiagen). We largely followed the sequencing protocol by Wahlberg and Wheat (2008), but PCR clean-up was carried out with ExoSAP-IT (Affymetrix, Santa Clara, CA, USA) and Sephadex columns (Sigma-Aldrich, St. Louis, MO, USA). Additionally, sequencing was performed using an ABI 3730 DNA Analyzer (Applied Biosystems, Foster City, CA, USA). Sequences were checked and edited using BioEdit software (Hall 1999). The sequences were uploaded to a VoSeq database (Peña and Malm 2012). The same dataset was used to generate fasta files for Neighbor-Joining analyses.

Minimum genetic p-distance barcode divergence between *M. laevigella* and *M. jussii* was calculated using analytical tools in BOLD Systems v. 4.0 (http://www.boldsystems.org). Neighbor-joining trees for the barcode region for all included *Monopis* species and specimens as well as four nuclear genes for five analyzed specimens of *M. laevigella* and *M. jussii* were constructed under p-distance model using Mega 7.0 (Kumar et al. 2016). The trees were stylized using CorelDraw v. 20.0.0.633.

## Results

**Figure 1.**
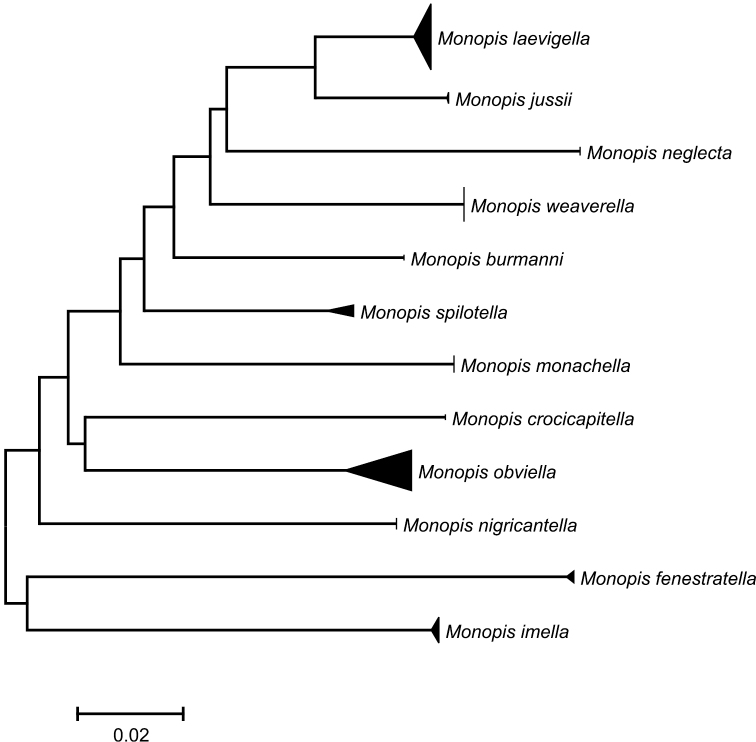
A compressed Neighbor-Joining tree DNA barcode region of European *Monopis* with most European species represented. The depth of the triangle is proportional to the intraspecific genetic variability within species and the height to sampling intensity.

DNA sequencing resulted in a barcode of 552 bp or longer for 81 specimens. All except seven specimens yielded a full-length (654 bp) barcode. BOLD’s barcode gap analysis showed that all included species have highly species-specific DNA barcodes with the mean of minimum divergences (p-distance model) to the nearest species being 10.01% (range 4.43–17.58%) (Figure 1). The minimum divergence between *M. laevigella* and *M. jussii* is 4.43%.

**Figure 2.**
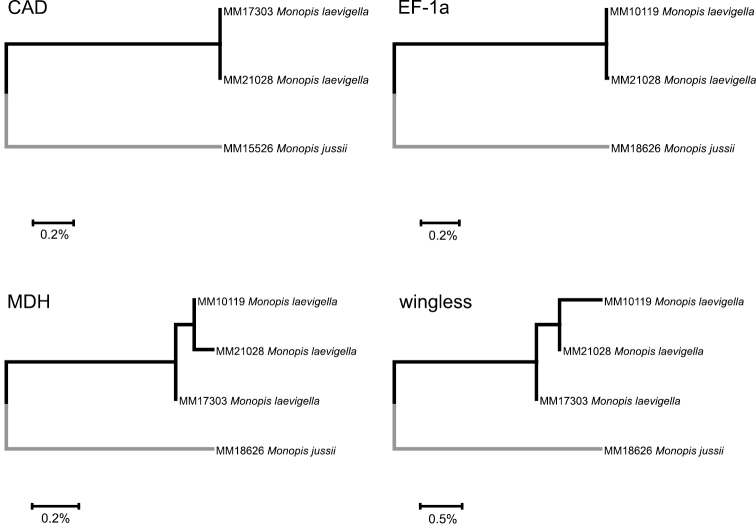
Comparison of genetic variability in four nuclear genes, CAD, EF-1a, MDH and wingless, between *Monopis laevigella* and *M. jussii* sp. nov.

For each nuclear gene, data of only a single specimen of two analyzed *M. jussii* specimens were retrieved. Informative (i.e. data from both species available) sequence lengths by genes were as follows: CAD: 336 bp, EF-1a: 410 bp, MDH: 334 bp, wingless: 307 bp. Genetic p-distances between the two species were: CAD: 2.1%, EF-1a: 2.2%, MDH: 1.5%, and wingless: 4.1%. As a rule, the specimen of *M. jussii* formed a sister to the two or three specimens of *M. laevigella* (Figure 2).

### Monopis jussii

Taxon classificationAnimaliaLepidopteraTineidae

Kaila, Mutanen, Huemer, Karsholt & Autto sp. nov.

BB76B4A7-8710-5482-9F0E-253DFB129B03

http://zoobank.org/288523EF-4785-4711-B5DF-483D42057841

**Figure 3.**
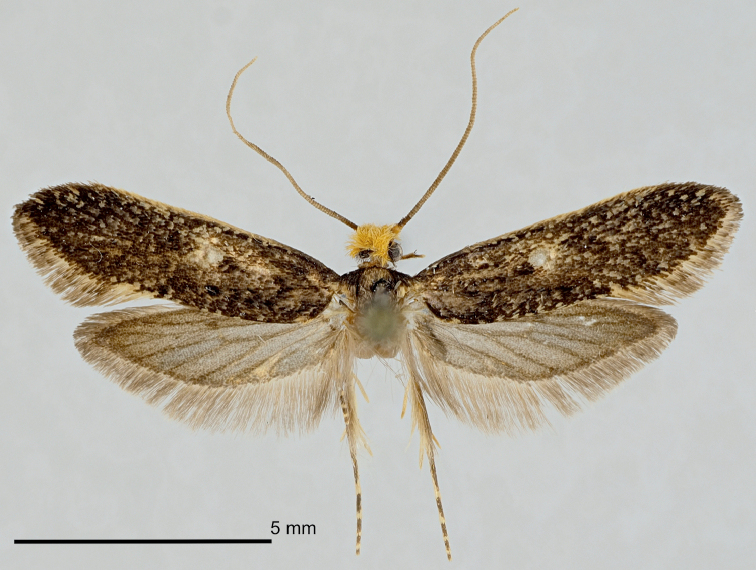
The holotype male of *Monopis jussii* sp. nov. PPe Yli-Kiiminki, larva 2001, ex nest of *Aegolius funereus*, M. Mutanen leg., R. Gaedike prep. 8607. (Coll. ZMUO).

**Figure 4.**
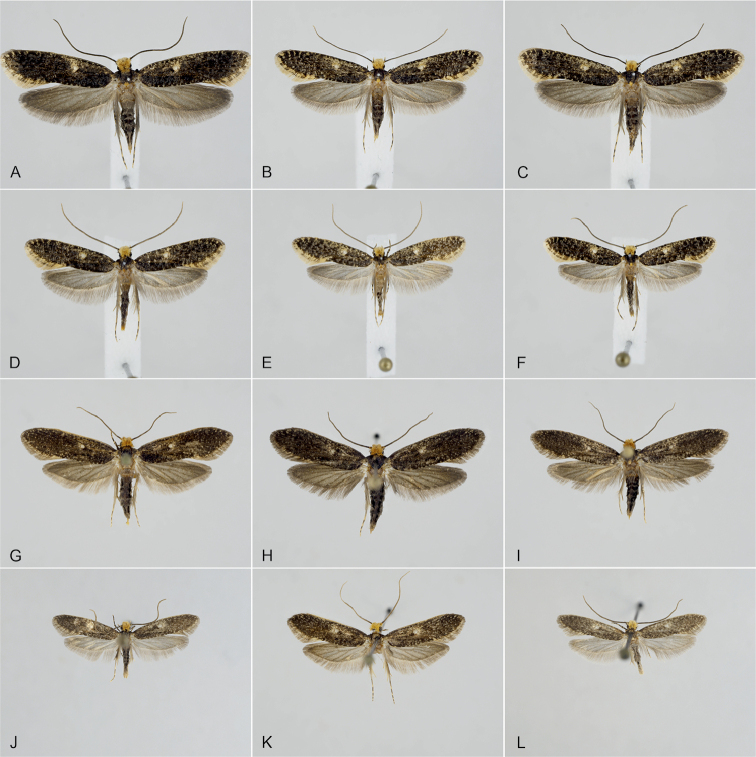
Comparison of habitus between *Monopis laevigella* and *M. jussii* sp. nov. **A–C***M. laevigella* female **D–F***M. laevigella* male **G–I***M. jussii* paratype, females **J–L***M. jussii* paratype, males.

**Figure 5.**
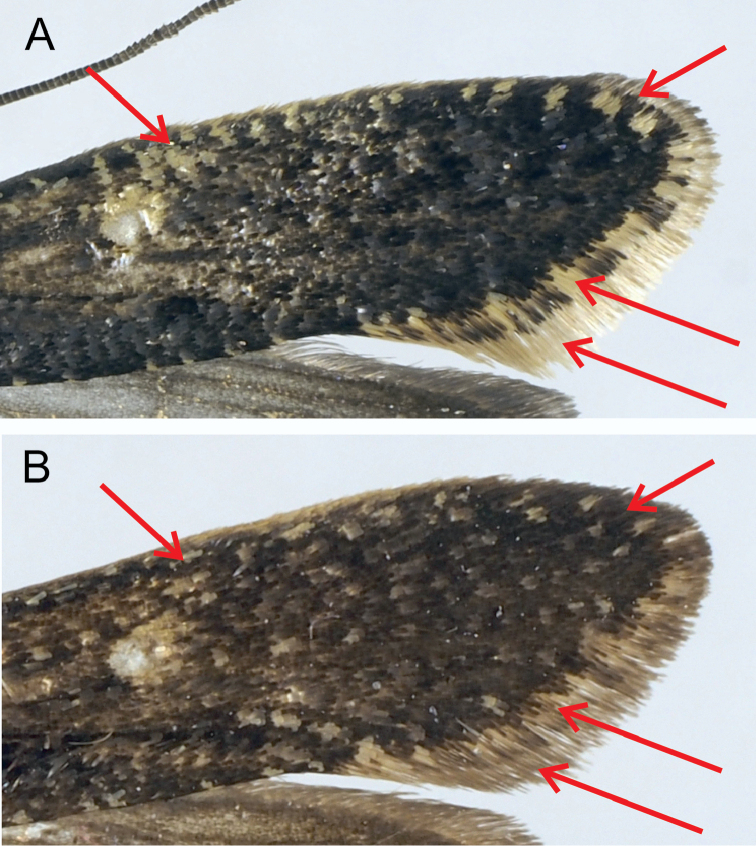
Comparison of forewing patterns of *Monopis laevigella* (**A**) and *M. jussii* sp. nov. (**B**). The arrows indicate differences in fringe colour (yellow/grey), fringe line (present/absent; chequered/non-chequered) and forewing costa (many white scales between the costa and the dorsal spot/few white scales between the costa and the dorsal spot).

**Figure 6.**
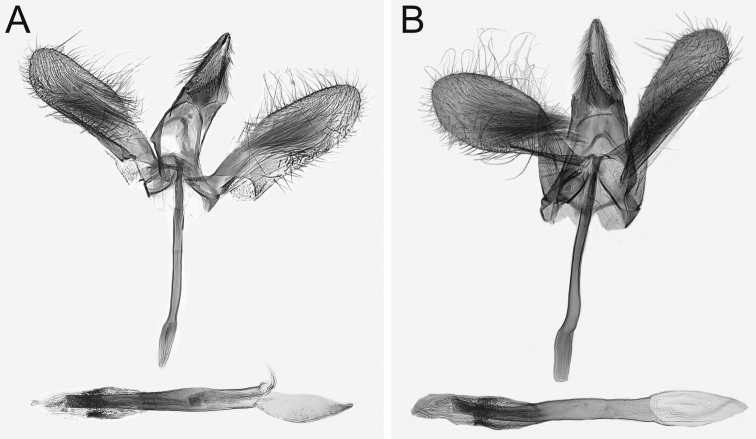
Overview of male genitalia of *Monopis jussii* sp. nov. **A** paratype, Finland, Kiiminki, M. Mutanen leg., L. Kaila prep. 6317 **B** Finland, Yli-Kiiminki, M. Mutanen leg., L. Kaila prep. 6315.

**Figure 7.**
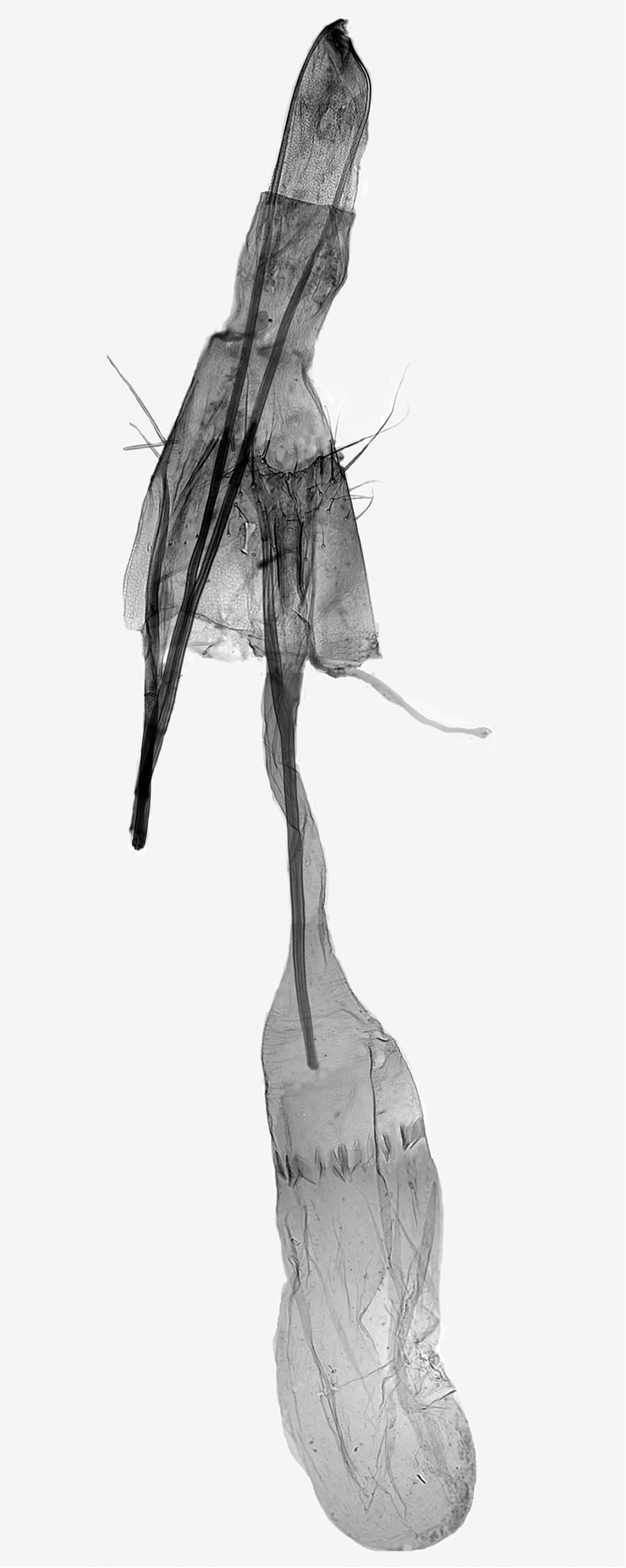
Overview of female genitalia of *Monopis jussii* sp. nov., paratype, Finland, Yli-Kiiminki, M. Mutanen leg., L. Kaila prep. 6324.

**Figure 8.**
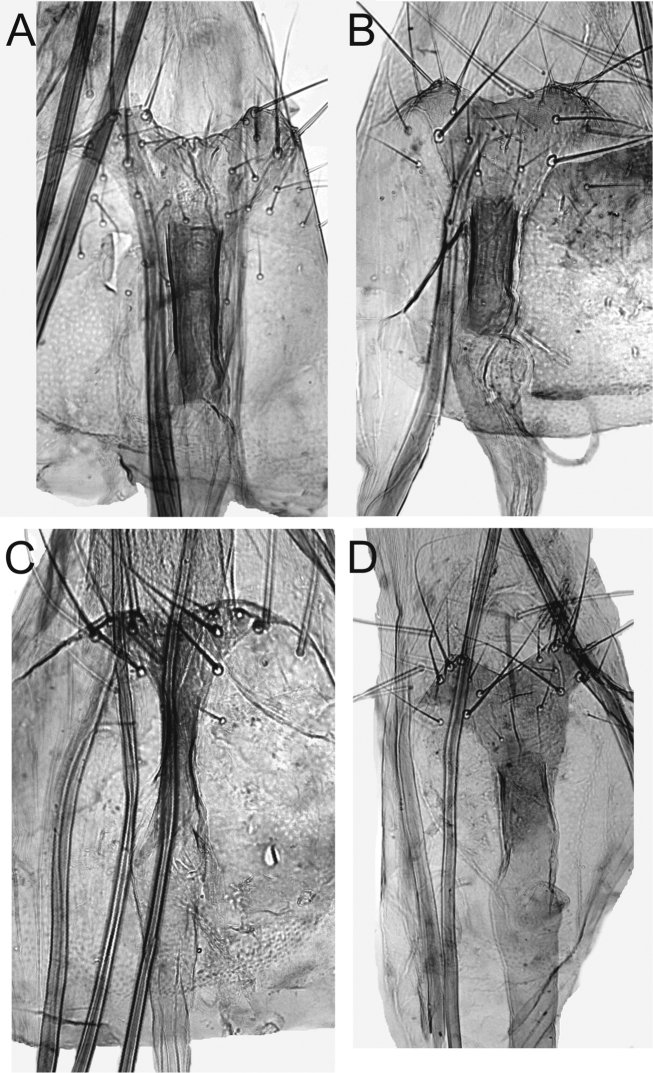
Details of ostium bursae and colliculum of female genitalia of *Monopis jussii* sp. nov. **A** paratype, Finland, Yli-Kiiminki, M. Mutanen leg., L. Kaila prep. 6324 **B** paratype, Finland, Kiiminki, M. Mutanen leg., L. Kaila prep. 6325 **C** paratype, Finland, Yli-Kiiminki, M. Mutanen leg., L. Kaila prep. 6322 **D** paratype, Finland, Kiiminki, M. Mutanen leg., L. Kaila prep. 6326.

**Figure 9.**
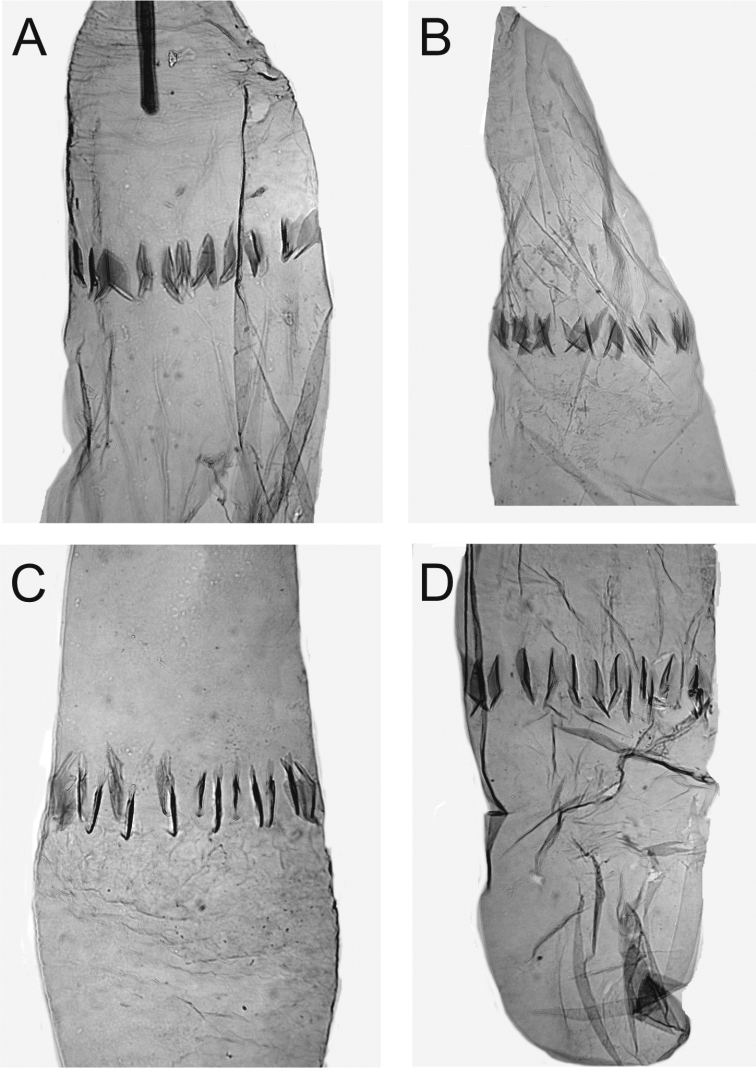
Signa of corpus bursae of female genitalia of *Monopis jussii* sp. nov. **A** paratype, Finland, Yli-Kiiminki, M. Mutanen leg., L. Kaila prep. 6324 **B** paratype, Finland, Kiiminki, M. Mutanen leg., L. Kaila prep. 6325 **C** paratype, Finland, Yli-Kiiminki, M. Mutanen leg., L. Kaila prep. 6322 **D** paratype, Finland, Kiiminki, M. Mutanen leg., L. Kaila prep. 6326.

Figures 3 , 4 , 5 , 6 , 7 , 8 , 9

#### Type material.

***Holotype*** ♂ (Figure 3): FINLAND, PPe Yli-Kiiminki, larva 2001, ex nest of *Aegolius funereus*, M. Mutanen leg. R. Gaedike prep. 8607. (ZMUO).

***Paratypes*.** FINLAND • 7 ♂ 16 ♀, PPs Kiiminki, 65.1163°N, 25.8291°E, Larva 1995, ex nest of *Aegolius funereus*, L. Kaila prep. 6317, 6325, 6326, M. Mutanen leg. (ZMUO); Finland: 10 ♂, 16 ♀, PPe Yli-Kiiminki, larva 2001, ex nest of *Aegolius funereus*, L. Kaila prep. 6314, 6315, 6316, 6322, 6323, 6324, R. Gaedike prep. 8606, 8607, 8698, DNA samples MM15526, MM17525, M. Mutanen leg. (ZMUO); • 2 ♀, Oba Utajärvi, Pälli, 64.8363°N, 26.21°E, larva 1980 ex nest of *Aegolius funereus*, J. Itämies leg. (ITJ); • 3 ♂ 3 ♀, Kn Puolanka, Piltunkijärvi, 64.7618°N, 27.3151°E, larva 18.6.1976 ex nest of *Aegolius funereus* (1974), M. Rikkonen leg. (ZMUO); • 2 ♂, Kn Vaala, Otermajärvi, 64.6724°N, 27.1047°E, larva 12 Jun 1976 ex nest of *Aegolius funereus* (1974), M. Rikkonen leg. (ZMUO); • 1 ♀, Kn Kajaani, 64.2263°N, 27.7932°E, VYÖ 1210 *ad luc* 15. –21 Jun 2006, DNA sample MM 17523, R. Leinonen leg. (ZMUO). ITALY • 1 ♀, Südtirol, Tiers E, Plafetscher Wald, 1600–1650 m, 46.472°N, 11.596°E, 23 Jun 2006, leg. Huemer, DNA sample TLMF Lep 09795 (TLMF).

#### Other material.

FINLAND • 7 ♂ 4 ♀, Ta Valkeakoski, Sääksmäki, 61.2326°N, 24.1137°E, ex larva (host unknown); 1992, S. Karhula leg. (MZH); • 2 ♀, Kn Kajaani, Karankalahti, 64.2222°N, 27.721°E, ex larva 2016 from nest of *Strix uralensis*, Itämies & Kyrki leg. (ZMUO); 1 ♀, PPe: Oulu, Oinaansuo, 65.0249°N, 25.6209°E, larva 28 Apr 1992 in nest of *Parus major*, J. Itämies leg. (ZMUO); • 1 ♀, EP Jurva, 62.7002°N, 22.0153°E, ex larva 2006, H. Vuorinen leg. (ZMUO); 2 ♀, Ks Kuusamo, 66.2565°N, 29.2807°E, ex larva 1975, J. Viramo leg. (ZMUO); • 1 ♂ 1 ♀, Ks Salla, Värriö, R1 & R3, 30 Jun 1989 & 21 Jul 1987, Erkki Pulliainen leg. (ZMUO); • 1 ♀, Li Inari, Kivijoki, 68.6125°N, 28.3509°E, 15 Jul 1993, E. & L. Laasonen leg. (ZMUO); • 1 ♂, Ks Kuusamo, Autiotalo, 66.3591°N, 29.6029°E, 28 Jun 1995, E. & L. Laasonen leg. (ZMUO); • 1 ♂, PPn Rovaniemi, 66.5509°N, 25.7619°E, 17 Jun 1992, T. Mutanen leg. (ZMUO); • 1 ♀, EnL Enontekiö, Saana, 69.0456°N, 20.8554°E, 11 Jul 2016, Marko, Nestori & Anttoni Mutanen leg. (ZMUO); • 1 ♀, Pedersöre, 8 Jul 1939, Sjöholm leg. (ZMUO); • 1 ♀, Om Jakobstad, 63.7098°N, 22.6489°E, 21 Jun 1936, E. Sjöholm leg. (ZMUO); 2 ♂, KP Haapajärvi, Harjunniemi, 63.7434°N, 25.3292°E, ad luc. 3 Jul 1975 & 6 Jul 1975, A. Kosonen leg. (ZMUO); NORWAY • Finnmark Alta, Mattisfossen-Sakkopadne, 5 Jul 1973, J. Kyrki leg. (ZMUO); SWEDEN • Härjedalen, Vemdalen, 3 Jul 1947, Henrik Bruun leg. (ZMUO).

#### Diagnosis.

*Monopis jussii* sp. nov. is externally close to *M. laevigella*, but the forewing appears darker, as it is less mottled with pale scales, especially along the margins (Figures 4, 5). Fringes are yellow and with a clear fringe line in *M. laevigella* but grey and without the fringe line in *M. jussii*. Besides the genetic markers, the forewing colour is indeed the best clue to separate these species. There is nevertheless some variation, especially in *M. laevigella*. Both male and female genitalia vary considerably, as do those of *M. laevigella*. The variation in all characters of genitalia overlaps between these species, and, apparently, they cannot be identified by genital characters. For variation of *M. laevigella* see also Gaedike (2019). Moreover, *M. weaverella* (Scott, 1858) and *M. neglecta* Šumpich & Liška, 2011 may occasionally fall within the morphological variation of these two species, especially in females. The males of *M. weaverella* and *M. neglecta* can however be distinguished from *M. laevigella* and *M. jussii* by the shape of gnathos, best decipherable in lateral view (see Gaedike 2019): gnathos arms are straight, triangular in *M. weaverella* and *M. neglecta*, angled particularly in anterior margin in *M. laevigella* and *M. jussii*.

#### Description.

Forewing length 5.8–8.5 mm (n = 8 ♂ and 8 ♀) (note that the specimens are reared which may have affected their size). Maxillary palpus, labial palpus and head ochreous yellow; outer side of labial palpus with dark grey scales, second segment distally bristled. Scape of antenna ochre with pecten formed of bristle-shaped scales, pedicel and flagellum dark brown. Thorax dark grey, dorsomedially variably intermixed or entirely with pale ochre scales; tegula dark grey, apically often paler grey or ochre. Fore and mid leg inwardly ochre, outwardly leaden grey, apex of tibia and tarsal segments ochre. Hind leg inwardly pale, outwardly ochre, intermixed with grey scales; spurs and apex of tibia and tarsal articles ochre. Forewing dark grey, variably mottled with pale grey scales; costa narrowly and variably sometimes ochre; basal scales of termen with alternating pale ochre and grey scales, distal scales of termen unicolorous grey, contrast between distally paler basal scales and darker distal scales giving an impression of faint fringe line; silvery grey spot somewhat basal of middle of wing length at fold. Hind wing bluish grey with somewhat darker grey veins; fringe basally narrowly ochre, otherwise grey. Underside of wings grey with ochre margin; underside of hindwing dark grey along costal margin. Abdomen leaden grey, basal segments ventrally more or less ochre.

***Male genitalia*** (Figure 6). Uncus elongate, triangular, laterally with long, hair-like scales, distally pointed, bifid. Gnathos arms angled in the middle, tapered toward hook-shaped apex. Basal and distal margins of tegumen reinforced, U-shaped, anterior margin more deeply. Shape of valva highly variable, gradually varying from ovoid and basally broadest to somewhat elongate and medially widest; distally round. Every aspect of saccus variable; straight or somewhat undulate, apically little or very much widened; length also very variable. Phallus straight and nearly parallel-sided, slightly widened at basal 1/3; length compared to that of saccus impossible to establish due to variation in length of saccus. Phallus distally inserted in cylindrical, internally spinose anellus. Vesica distally densely spinose, devoid of cornuti.

***Female genitalia*** (Figures 7–9). Papilla analis membranous, elongate, distally round, with a few setae. Apophysis posterioris as long as segments 7+8, posteriorly starting as continuation of papilla analis, slender, anteriorly slightly widened, apex cut. Apophysis anterioris 1/3 length of and slightly stouter than apophysis posterioris, twice as long as 8^th^ segment, distally not widened. Ovipositor telescopic, with two retractile nodes; with a few stout setae. Ventral pseudapodemes (*sensu*Davis and Robinson 1999) not decipherable. Tergum 8 posteriorly somewhat sclerotized. Ostium a widely U-shaped opening, laterally bordered as posteriorly curved rim, laterad shallowly emarginated in posterior direction, emargination with a few long setae; devoid of microtrichia but minutely granulose. Length of antrum variable, narrowed toward colliculum; colliculum tubular, length variable, 2–4 times as long as wide, usually narrowed in the middle. Ductus bursae between colliculum and corpus bursae membranous, as long as apophysis anterioris. Corpus bursae oval, 3 times as long as wide; in approximately the middle to posterior 1/3 ca. 12 elongate, sharply spicular or dentate signa forming transverse band.

#### Genetic characterisation.

Clearly distinguishable by its DNA barcode from all other species of *Monopis* barcoded globally so far (Figure 1). Genetically the closest species with a minimum divergence of 4.43% is *M. laevigella*. Intraspecific divergence among four barcoded specimens from Finland and Italy is 0.15%. Additionally, the species show 1.5–4.1% interspecific divergence in the nuclear genes of *CAD*, *EF-1a*, *MDH* and *wingless* (Figure 2).

#### Etymology.

The species is dedicated to Dr Juhani (Jussi) Itämies, a Finnish expert of Lepidoptera who, as far as we know, is the first to have reared this species. He has also spent most of his life on faunistic research of Finnish Lepidoptera and has done incredible work in elucidating the life history of numerous microlepidopteran species.

#### Distribution.

From our available observations *M. jussii* seems to have a boreo-montane distribution pattern. It is widely distributed in Finland and also recorded from Norway (Finnmark) and Sweden (Härjedalen). Records from the Alps seem rare with a proved, barcode-based locality in the Italian Dolomites and two further unpublished records (ZSM, A. Segerer) in the Bavarian Alps.

#### Biology.

So far reared on five different occasions from the nest bottoms of the Boreal owl (*Aegolius funereus*). Two specimens in the collection of ZMUO have been reared from the nest of the Ural owl (*Strix uralensis*) and one specimen from the nest of the Great tit (*Parus major*). Additionally, three reared specimens of two different rearing events do not state anything about the origin. One specimen has been found in a vacated house. Thirteen specimens in coll. ZMUO and a specimen from the Italian Alps in coll. TLMF have been collected in the wild between 17 June to 21 July, which matches well with the flight time of other *Monopis* species of these regions.

##### Taxonomic remarks on *Monopis laevigella*

*Monopis jussii* sp. nov. is most closely related to *M. laevigella* and can easily be confused with that species (see above). We therefore re-evaluate available names in the *M. laevigella* species group.

*Monopis laevigella* ([Denis & Schiffermüller], 1775).

*Tinea laevigella* [Denis & Schiffermüller], 1775: 139.

##### Misidentifications

*Tinea rusticella* Hübner, 1796: 61, pl. 3, fig. 17; a junior synonym of *Haplotinea insectella* (Fabricius, 1794) (Zeller, 1852: 153–154).

*Recurvaria rustica* Haworth, 1828: 548; unjustified emendation of *Tinea rusticella* Hübner, 1796.

*Tinea saturella* Haworth, 1828: 562, unavailable.

*Tinea vestianella* sensu Stephens, 1835: 344; a misidentification of Phalaena (Tinea) vestianella Linnaeus, 1758.

Blabophanes rusticella ab. semispilotella Strand, 1900: 225; unavailable name, deemed infrasubspecific according to ICZN Art. 45.6.2 from use of the term “ab.”; a misidentification of *M. weaverella* (Scott, 1858) (Gaedike 2019).

##### Neotype selection

**Figure 10.**
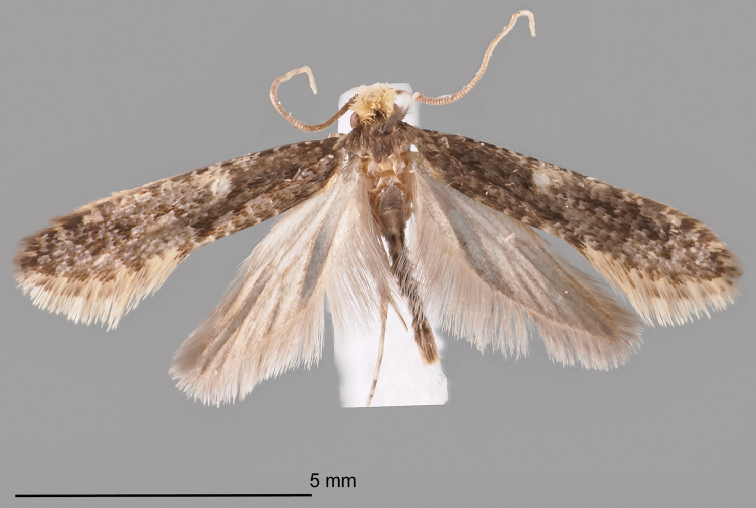
Neotype male of *Monopis laevigella* from Austria, here designated. AUSTRIA occ. Nordtirol / Brandenberg / Tiefenbachklamm / 11°51'52"E, 47°29'4"N / 645 m, 16.6.2013 / leg. Huemer” “DNA Barcode / TLMF Lep 10354”. (Coll. TLMF).

*Tinea laevigella* was described from an unspecified number of specimens collected in the area of Vienna, Austria ([Denis & Schiffermüller], 1775). The collection was later deposited in the “Hof-Naturalien-Kabinett” and destroyed by fire during the Vienna Rebellion on 31^st^ of October 1848 (Speta 2003). Since this species can be confused with *M. jussii* sp. nov. and several other congeneric taxa we designate as neotype a male specimen from Austria to preserve stability (Figure 10). It is labelled “AUSTRIA occ. Nordtirol / Brandenberg / Tiefenbachklamm / 11°51'52"E, 47°29'4"N / 645 m, 16.6.2013 / leg. Huemer” “DNA Barcode / TLMF Lep 10354” (TLMF).

*Tinea rusticella* was figured twice by Hübner in the eighth volume of his *Sammlung europäischer Schmetterlinge*, first it was validly described on page 61, pl. 3, fig. 17 (1796) and later a different species was figured on pl. 49, fig. 339 (1813). Hübner (1825) considered them conspecific, and he referred to both figures when he erected the monotypic genus *Monopis*.

Zeller (1852) was probably the first to question whether Hübner’s two figures of *Tinea rusticella* represented the same species. He referred to Hübner’s fig. 339 (1813) when dealing with the species, which became known as *Monopis rusticella* [= *Monopis laevigella* ([Denis & Schiffermüller], 1775)], and rejected that Hübner’s fig. 17 (1796) could be of a specimen of that species, suggesting that it could be *Tinea misella* Zeller, 1839 [= *Haplotinea insectella* (Fabricius, 1794)]. *Tinea rusticella* Hübner, 1813 is both a misidentification and a homonym of *Tinea rusticella* Hübner, 1796 and thus permanently invalid.

Haworth (1828: 548) named the species twice. First with reference to Hübner’s pl. 3, fig. 17 as *Recurvaria rustica*, which is an unjustified emendation and thus an objective synonym of *Tinea rusticella* (Hübner, 1796) [= *Haplotinea insectella* (Fabricius)], and later in the same work Haworth (op. cit.: 339), again with reference to Hübner’s pl. 3, fig. 17, proposed the name *Tinea saturella* in synonymy with *Tinea rusticella*. Because *Tinea saturella* was described in synonymy with *Tinea rusticella* it was always considered a synonym of that species (viz. *Monopis rusticella*), but because Haworth referred only to Hübner’s fig. 17 (and not to fig. 339) it is an objective junior synonym of *Tinea rusticella* Hübner, 1796, and thereby a subjective junior synonym of *Haplotinea insectella* (Fabricius). However, as the name *Tinea saturella* has never been made available under the provision of Art. 11.6. of the Code (ICZN 1999) and adopted as the name of a taxon before 1961, we consider it as unavailable.

Although *Monopis* Hübner 1825 was described as a monotypic genus, it is based on a partly misidentified species. We consider Zeller (1852) as First Reviser of *Tinea rusticella* Hübner, restricting the name to the species now (and also by Zeller 1852) known as *Monopis laevigella* ([Denis & Schiffermüller], 1775).

## Discussion

Compared with many other groups of Lepidoptera, the species diversity of Tineidae is generally poorly investigated. Hundreds of species deposited in museum collections remain undescribed (Robinson 2009). It is likely that many more species remain entirely undiscovered globally. The European fauna is comparatively well understood, and the fauna of the entire continent has recently been taxonomically reviewed in two monographs (Gaedike 2015, 2019). New species discoveries are uncommon, particularly for central and northern parts of Europe. An example of a recent species discovery is that of *Monopis neglecta* Šumpich & Liška, 2011, a species that morphologically is nearly indistinguishable from *M. weaverella* (Scott, 1858) (see Gaedike 2019). While no genetic data were provided for *M. neglecta* in the original description, the DNA barcode sequences provided in the present study confirm its status as a separate species from *M. weaverella*. It is encouraging that although the species of Tineidae are often difficult to tell apart from each other morphologically, no cases of barcode sharing in the European fauna are known. Evidently, therefore, DNA barcoding provides an efficient way to investigate their diversity in less thoroughly explored areas as well.

Based on the available distributional data, *Monopis jussii* has a much more limited range than *M. laevigella*. It is possible, if not likely, that it is a member of boreo-montane faunal elements, being distributed in the boreal region on the one hand and in the Alps below the timberline on the other hand. It is likely absent from the lowlands of Central Europe. It would not be surprising if the species turns out to be present in other European mountain systems and the eastern Palearctic. Based on the large number of examined museum specimens from the ZMUO and MZH collections, the species is widely present in northern Finland south to ca. 64° N but becomes much scarcer towards the more southern localities. The southernmost verified records from Finland are from the province of Tavastia australis (ca. 61° N).

Based on our own and other experiences (Robinson 2009, Gaedike 2019), *Monopis laevigella* is not strict regarding the source of its food, but it seems to prefer cavity-breeding birds, possibly because their nests are usually dry. Several extensive rearing experiments of nest bottoms of various birds, mostly the Tawny Owl (*Strix aluco* Linnaeus, 1758) and the Ural Owl (*S. uralensis* Pallas, 1771), from southern Finland have yielded large numbers of *M. laevigella*, which is usually present in every nest in large numbers. In an experiment by MM in 2017 with 13 nest bottoms of *Strix* spp., probably thousands of *M. laevigella* emerged. Among several dozen pinned specimens sampled from each nest, none represents *M. jussii*. Other species that are regularly or often present in the nests of *Strix* spp. in Finland are *Niditinea striolella* (Matsumura, 1931) (usually emerges in great numbers too), *Tinea svenssoni* Opheim, 1965 (present in almost all nests), *Tinea steueri* Petersen, 1966 (not present in every nest) and *Monopis fenestratella* (Heyden, 1863) (present in most nests but is cryptic in behaviour). While it is possible that *M. jussii* has stricter habitat requirements and that it has a strong preference for the Boreal Owl, we find this possibility unlikely. The Boreal owl, the Ural owl, as well as the Great tit are all cavity breeders, rendering the nest conditions between these species very similar. In rearing conditions, tineids are not selective for the origin of food and readily feed on mammal hairs too. It is more likely that *Monopis jussii* has been reared mostly from the nests of the Boreal owl just because it is a more common owl species within the moth’s main distribution in Finland than either of the two *Strix* species present in Finland. Further rearing experiments, optimally systematically from different species of birds, would bring additional valuable information on the habitat requirements of *M. jussii* and several other species of Tineidae.

*Monopis laevigella* has a Holarctic distribution (Landry and Pohl 2018, Gaedike 2019). Many specimens of this species have been barcoded from the Nearctic region, both from Canada and the U.S.A. They fall in two clusters, both of which are highly distinct from the clade consisting of *M. jussii* and the Palearctic *M. laevigella* (data only partially public in BOLD). In the Neighbor-Joining trees neither of these clusters is placed as sister to the Palearctic *M. laevigella* + *M. jussii* clade, suggesting that they represent distinct taxa and even that their closest relative is not *M. laevigella*. However, due to the limited phylogenetic information content of the DNA barcode region, verification of both scenarios requires more rigorous and thorough taxonomic and phylogenetic scrutiny.

## Supplementary Material

XML Treatment for Monopis jussii
